# The Associations Between the Religious Background, Social Supports, and Do-Not-Resuscitate Orders in Taiwan: An Observational Study

**DOI:** 10.1097/MD.0000000000002571

**Published:** 2016-01-22

**Authors:** Kuan-Han Lin, Yih-Sharng Chen, Nai-Kuan Chou, Sheng-Jean Huang, Chau-Chung Wu, Yen-Yuan Chen

**Affiliations:** From the Graduate Institute of Medical Education and Bioethics, National Taiwan University College of Medicine (K-HL, C-CW, Y-YC); and Department of Surgery, National Taiwan University College of Medicine, Department of Surgery, National Taiwan University Hospital, Taipei, Taiwan (Y-SC, N-KC, S-JH).

## Abstract

Prior studies have demonstrated important implications related to religiosity and a do-not-resuscitate (DNR) decision. However, the association between patients’ religious background and DNR decisions is vague. In particular, the association between the religious background of Buddhism/Daoism and DNR decisions has never been examined. The objective of this study was to examine the association between patients’ religious background and their DNR decisions, with a particular focus on Buddhism/Daoism.

The medical records of the patients who were admitted to the 3 surgical intensive care units (SICU) in a university-affiliated medical center located at Northern Taiwan from June 1, 2011 to December 31, 2013 were retrospectively collected. We compared the clinical/demographic variables of DNR patients with those of non-DNR patients using the Student *t* test or *χ*^2^ test depending on the scale of the variables. We used multivariate logistic regression analysis to examine the association between the religious backgrounds and DNR decisions.

A sample of 1909 patients was collected: 122 patients had a DNR order; and 1787 patients did not have a DNR order. Old age (*P* = 0.02), unemployment (*P* = 0.02), admission diagnosis of “nonoperative, cardiac failure/insufficiency” (*P* = 0.03), and severe acute illness at SICU admission (*P* < 0.01) were significantly associated with signing of DNR orders. Patients’ religious background of Buddhism/Daoism (*P* = 0.04), married marital status (*P* = 0.02), and admission diagnosis of “postoperative, major surgery” (*P* = 0.02) were less likely to have a DNR order written during their SICU stay. Furthermore, patients with poor social support, as indicated by marital and working status, were more likely to consent to a DNR order during SICU stay.

This study showed that the religious background of Buddhism/Daoism was significantly associated with a lower likelihood of consenting to a DNR, and poor social support was significantly associated with a higher likelihood of having a DNR order written during SICU stay.

## INTRODUCTION

The American Heart Association first approved the clinical use of cardiopulmonary resuscitation (CPR) in 1974, and also proposed the concept of “Do-Not-Resuscitate” (DNR) by addressing that decisions not to resuscitate should be recorded in the medical charts and communicated to all the medical staff.^1^ In 1976, 2 healthcare institutions in the United States first reported their process on how the appropriateness of DNR for a patient is determined.^2,3^ Recognized by the President's Commission for the Study of Ethical Problems in Medicine,^4^ encouraged by the Joint Commission requirements on hospital DNR policy,^5^ and endorsed by the Patient Self-Determination Act passed in 1990, DNR now is frequently discussed in healthcare institutions in the United States.

Three retrospective, observational, and single-center studies using medical records review reported that the prevalence of DNR orders varied from 9.3% to 41%.^6–8^ The factors shown to be related to DNR decisions were older age,^7,9–11^ white race,^7,12^ and more severe illnesses at admission.^7,13^ In addition, several studies have been focused on the medical care DNR patients received after the orders were written. They have reported that DNR decisions are linked to fewer life-supporting treatments (LSTs), a lower likelihood of physiologic monitoring, and a lower likelihood of being admitted to intensive care units (ICUs).^14–18^

Karches et al^19^ reported that patients’ self-reporting high intrinsic religiosity was associated more with specifying a surrogate decision-maker than those who self-reported low intrinsic religiosity, but was not significantly associated with having a DNR order. Family members of patients who perceived themselves as religious were more likely to oppose the physicians’ recommendation of implementing DNR for the patient.^20^ A study conducted by Hileli et al^21^ in Israel showed that religious background did not have a significant association with a DNR decision when the religious background was categorized as Jewish, Muslim, Christian, or Druze.

DNR has been a part of the clinical practice in Taiwan for decades. Legal immunity for not resuscitating imminently dying patients was provided to healthcare professionals after the Taiwanese lawmakers established the Hospice-Palliative Care Act in 2000.^22^ Decisions about DNR have to be made by patients, or surrogate decision-makers (usually the family members) for incompetent patients, in consultation with their physicians. Several studies conducted in Taiwan have reported that older age,^23–25^ unmarried,^24^ and longer stay in an ICU ^25^ were associated with a DNR decision.

Prior studies have demonstrated important implications in relation to religiosity and a DNR decision, and only some of them reported the association between patients’ religious backgrounds and DNR decisions. Nevertheless, neither the studies conducted around the world nor in Taiwan have examined the association between Buddhism/Daoism, one of the major religions in the world as well as in Taiwan, and a DNR decision in clinical practice. Obtaining the knowledge of all components of the patient's background and their relationships to the DNR decision will lead to optimal medical care. A lack of understanding of the religious background of the patient or the family members may render difficulty in optimally valuing frameworks and preferences in making medical decisions.^26^

This study aimed to examine the association between patients’ religious background and their DNR decisions, with a particular focus on Buddhism/Daoism, after controlling for other confounding variables.

## METHODS

This is a retrospective observational study conducted in the surgical intensive care units (SICUs), 2 cardiovascular SICUs and 1 thoracic SICU, in a medical center located in northern Taiwan. The medical records of the patients who were at the age of 18 or older, admitted for the first time to 1 of the 3 SICUs between June 1, 2011 and December 31, 2013, and with a Therapeutic Intervention Scoring System (TISS) score recorded upon SICU admission were reviewed. This study was approved by the Research Ethics Committee in National Taiwan University Hospital (20140308RINC). Because this is an observational study with medical records review, written informed consent from the study participants was waived. The methods of conducting this study, as well as keeping the confidentiality for the dataset, were in conformity to those approved by the Research Ethics Committee.

The following variables were collected: age, sex, educational level, working status, marital status, severity of illness upon SICU admission as indicated by the TISS score, length of hospital stay, length of SICU stay, SICU admission diagnosis, DNR status, the interval between SICU admission and a DNR order written, the interval between a DNR order written and SICU discharge, and religious background categorized as Buddhism/Daoism, Christian/Catholics, and others.

TISS was developed by Cullen et al^27^ in 1974. It has become a widely accepted measure for indicating the severity of illness. This is done by applying points from 1 to 4, to a list of 76 therapeutic items. The total score is calculated by summing the scores derived from each therapeutic item. The scores range from 0 to 174 points. Higher scores of TISS indicate more severe illness and require a higher number of therapeutic interventions, and vice versa.

The SICU admission diagnosis was chosen from the 50 APACHE II (Acute Physiology and Chronic Health Evaluation II) diagnostic categories.^28^ We collapsed the 50 diagnostic categories into only 4 categories: nonoperative, cardiac failure/insufficiency; nonoperative, others; postoperative, major surgery; and postoperative, others.

All statistical analyses were conducted using SAS 9.2 (SAS Institute Inc, Cary, NC). Patients were classified into 2 groups according to their status of DNR during SICU stay. Those who had a DNR order written during their SICU stay were considered to be in the “DNR” group. The other patients were assigned to the “non-DNR” group. The differences in demographic data between the DNR group and the non-DNR group were examined using Student *t* test and *χ*^2^ test depending on the scale of the 2 variables. Multivariate logistic regression analysis was then performed to assess the relationship between patients’ religious background and their DNR decision after controlling for other variables.

The deliberation of the multivariate logistic regression model was examined using the area under the receiver operating characteristic curve based on the simultaneous measure of sensitivity (true positive) and specificity (true negative) for all possible cutoff points.^29^ The calibration of the model was examined using the Hosmer and Lemeshow Goodness-of-fit test.^30^ A *P* value of less than or equal to 0.05 was considered statistically significant.

## RESULTS

**FIGURE 1:**
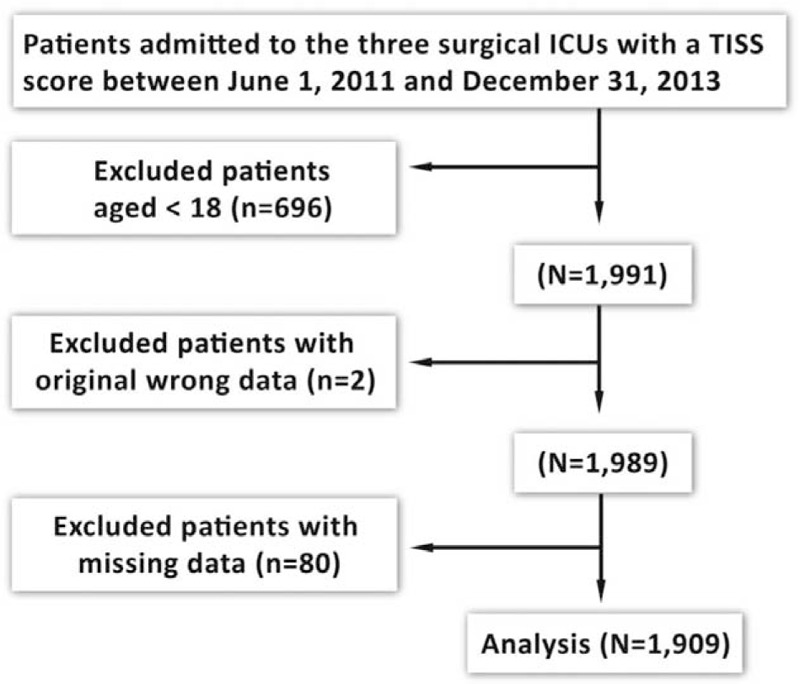
Enrollment of the patients in this study.

Two thousand six hundred eighty-seven patients were admitted to the SICUs during data collection period. Among them, 696 were excluded because they aged younger than 18, 2 for administratively wrong data, and 80 for a missing value in any collected variables. A total of 1909 patients were included in the final analysis (Figure 1). Among the 1909 patients, 122 (6.4%) had a DNR order written during their SICU stay.

There were 1285 male patients (67.3%) and 624 female patients (32.7%), with a mean age of 61.5 years. Buddhist/Daoist accounted for approximately 45% of the research subjects. Most patients were married (76.2%), and approximately 30.4% of them had an educational level of college/university or above. The mean TISS of the 1909 patients was 32. The average length of stay in the SICUs and in the hospital was 6.4 and 24.4 days, respectively. Compared with the patients in the non-DNR group, the DNR group was significantly older (*P* *<* 0.05), had higher severity of illness at SICU admission (*P* < 0.01), and had longer length of SICU (*P* < 0.01) and hospital (*P* = 0.02) stay (Table 1).

**TABLE 1:**
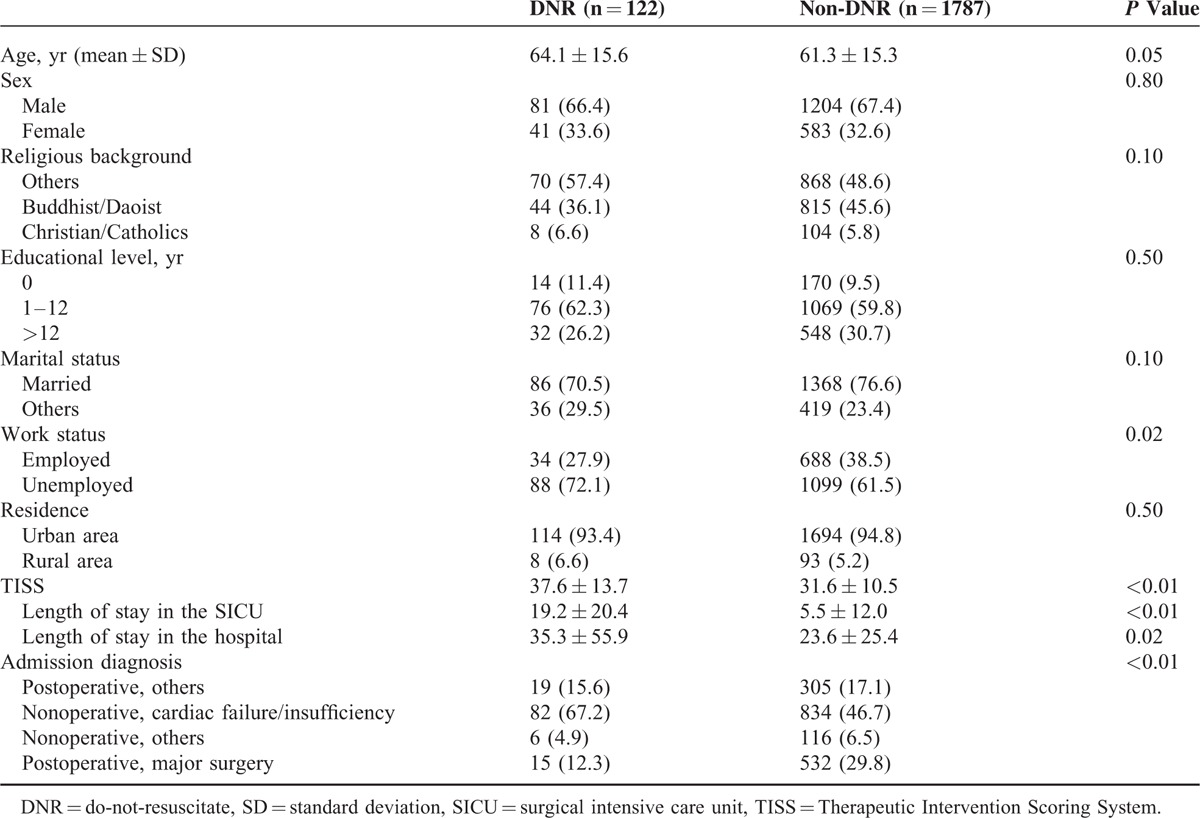
Demographic and Clinical Characteristics of the DNR and Non-DNR Patients

The most prevalent admission diagnosis was related to cardiac disorders, for example, congestive heart failure, coronary artery disease, sepsis, cardiogenic shock, and dissecting aortic. About 48% of the 1909 patients (67% in the DNR group and 47% in the non-DNR group) were admitted to the SICUs because of “nonoperative, cardiac failure/insufficiency” (Table 1).

The results of the multivariate logistic regression analysis are shown in Table 2. Old age (*P* = 0.02), unemployment (*P* = 0.02), admission diagnosis of “nonoperative, cardiac failure/insufficiency” (*P* = 0.03), and severe acute illness at SICU admission (*P* < 0.01) were significantly associated with signing of DNR orders after controlling for other confounding variables. Moreover, patients’ religious background of Buddhism/Daoism (*P* = 0.04), married marital status (*P* = 0.02), and admission diagnosis of “postoperative, major surgery” (*P* = 0.02) were less likely to have a DNR order written during their SICU stay.

**TABLE 2:**
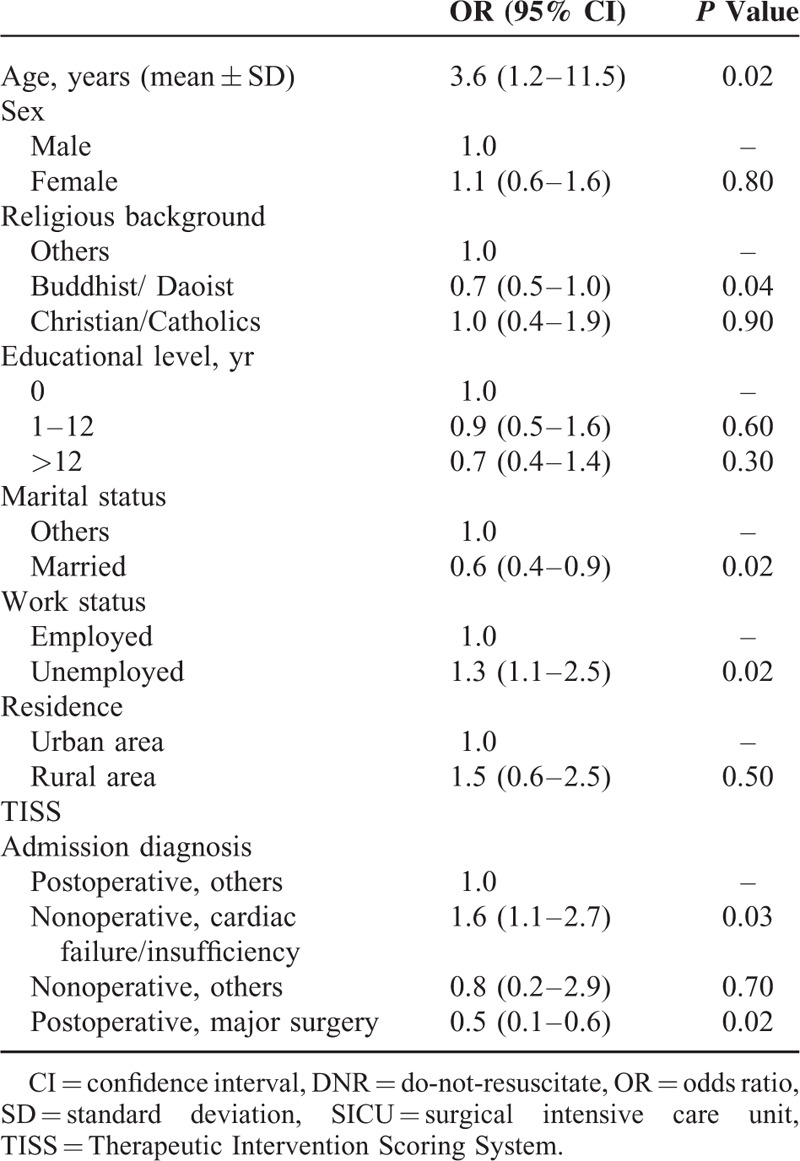
Factors Associated With the Signing of DNR Orders

The area under receiver operating characteristic curve was 0.71 (95 % CI = 0.67–0.76, *P* =  < 0.01), indicating acceptable deliberation of the multivariate logistic regression model. The *P* value of the Hosmer and Lemeshow Goodness-of-fit test was 0.6, not rejecting the null hypothesis that the model has a good fit, indicating the good fitness of this model.

## DISCUSSION AND CONCLUSIONS

### Main Outcome

In this study, we retrospectively reviewed 1909 SICU patients’ medical records. Among them, 6.4% of the patients had a DNR order written during their SICU stay. The results of this study showed that old age, unemployment, admission diagnosis of “nonoperative, cardiac failure/insufficiency,” and severe acute illness at SICU admission were positively associated with consenting to a DNR order. In comparison, a religious background of Buddhism/Daoism, married marital status, and an admission diagnosis of “postoperative, major surgery” were negatively associated with a DNR order written.

### Generalizability

Phillips et al,^31^ based on a cohort derived from the Study to Understand Processes and Preferences for Outcomes and Risks of Treatments SUPPORT, reported that older age and severity of illness are the predictors for DNR decisions in ICUs after controlling for other confounding variables. According to a multicenter study conducted in 68 medical centers in the United States by Nathens et al,^11^ 6.86% of the 6,765 patients had a DNR order written. Independent patient characteristics associated with a DNR decision were older age and more severe clinical illness. Chen et al^7^ conducted a single-center study in Northeast Ohio, also reported that older age, severity of illness at admission to the medical ICU, and race/ethnicity were significantly associated with a DNR order written during ICU stay.

Similar to several prior DNR studies, our study also found that 6.4% of the 1909 patients admitted to the SICUs collected during the study period had a DNR order written during their stay in the SICU. In addition, increasing age and more severe clinical illness at admission predicted patients’ DNR decisions. The generalizability of the results of this single-center study is as good as several prior studies as indicated by the percentage of patients who had a DNR order written, age, and severity of illness upon SICU admissions.^7,11,31^

### Do-Not-Resuscitate and Religious Background

According to a study on the world's major religious groups reported by the Pew Research Center, there are about 488 million Buddhists and 8 million Daoists worldwide, representing 8% of the world's total population as of 2010.^32^ The most important religions in Taiwan are Buddhism and Daoism, which are practiced by the majority of the population. The Department of Civil Affairs, Ministry of the Interior of Taiwan in 2005 reported that the total population of Buddhists and Daoists in Taiwan is about 16 million, accounting for approximately 68% of Taiwan's population.^33^

Confucianism, Buddhism, and Daoism are the most essential part of the traditional Chinese culture. Deeply influenced by the traditional Chinese culture, Confucianism, Buddhism, and Daoism constitute the essence of the Taiwanese culture, as well as the Taiwanese religions. Therefore, when patients or their surrogates encounter end-of-life decision-making, the influences of a mixture of different religious backgrounds or philosophical beliefs may contribute to the decision-making process, and may end up with the various opinions and dilemmas. This study found that the patients with the religious background of Buddhism/Daoism were less likely to consent to a DNR order. Several explanations may account for this phenomenon:

First, filial piety, highlighted by Buddhism in Taiwan, may be associated with a lower likelihood of consenting to DNR. In Taiwan, either patients or surrogates can consent to a DNR order after sufficient communication with health care professionals. Nevertheless, the majority of end-of-life decisions in Taiwan, for example, DNR, withholding artificial nutrition, withdrawing mechanical ventilation, and so on, is made by surrogate decision-makers.^23^ The patients’ family members who are surrogate decision-makers may consent to LSTs for the patients without carefully deliberating the patients’ preferences.^34^ Instead, the surrogate decision-makers may consent to DNR based on their personal preferences influenced by their religious backgrounds, religiosity,^19^ race/ethnicity,^35,36^ information given by significant others,^37^ and so on.

Filial piety has been supported by Buddhism since it was introduced to China.^38^ The social norm formed by filial piety provides the expectation that offspring should take care of their parents and should extend parents’ lives to the last minute.^38^ As such, the offspring with filial piety are less likely to consent to DNR because “not doing CPR” may be considered not taking care of parents, and not extending their lives as long as possible. In addition, once the offspring decided to forgo LSTs, they might be blamed by the society and their relatives for not taking responsibility of saving their parents’ lives, and for not complying with filial piety.^39,40^ Even if the family members do not have clear understandings on LSTs, they may still be reluctant to make the decision of discontinuing LSTs for their parents.^41^

Second, the teachings of karma may play an important role in not consenting to DNR. Although Buddhists accept withdrawing of LSTs for patients at the end of life,^42^ the decision-making process of signing a DNR order may be unpredictable due to different perspectives on encountering the event of death. For example, if the patient/surrogate decision-maker believes in the teachings of karma^43^ (ie, a good intention and a good act lead to good karma and future happiness, and a bad intention and a bad act lead to bad karma and future unhappiness^44^), and see the sufferings of death as a process to gain good karma and happiness in the rebirth, they may not easily forgo LSTs and not consent to DNR.

Third, the doctrine of Daoism may also attribute to a lower likelihood of consenting to a DNR order. Daoists believe in an afterlife of torture and endless suffering of hell. The teachings of Daoism emphasize the maintenance of youth and achievement of longevity and immortality to avoid the torture and suffering in hell.^45^ Patients with a strong religious background of Daoism may resist inevitable death and pursue life extension as long as possible, thus not consenting to DNR.

### Do-Not-Resuscitate and Social Support

This study also showed that the work status of unemployment (*P* = 0.02), and other marital status (*P* = 0.02) such as unmarried, divorced, and widowed were significantly associated with consenting to DNR order. The associations shown in this study may be attributed to the lack of social support for the patient.

Social support is defined as being able to access family members and friends that a person can rely upon if he/she needs. Several studies have pointed out that people with a spouse and friends have better health than those without a spouse and friends .^46^ Social support has been reported to be associated with better outcomes for people with medical conditions.^47,48^

Decisions to consent to DNR orders in Taiwan are usually made by family members as surrogate decision-makers.^23,49,50^ Those who were designated as surrogate decision-makers for the patients, supposed to have poor social support as indicated by unemployment, unmarried/divorced/widowed marital status, may not clearly understand the preferences and best interests of the patients they are representing. The designated surrogate decision-makers may easily consent to DNR as long as they are consulted by healthcare professionals for DNR, without carefully deliberating the preferences and best interests of the patient. As a result, patients with poor support as indicated by work and marital status in this study were more likely to have a DNR order consented to by their surrogate decision-makers.

### Strengths and Limitations

Prior studies have examined the association between religiosity and a DNR decision, as well as the association between the religious background of Christians/Catholics and a DNR decision. Our study further adds to the literature of DNR by examining the association between the patients’ religious background of Buddhism/Daoism and DNR decisions. In addition, our study examined the association between social support and DNR.

There are several limitations in this study. The first limitation is the generalizability of the results of this study. This is a single-center study conducted in 3 SICUs, and it is possible that our findings do not reflect the situation existing in other ICUs or other healthcare institutions.

Second, since we used retrospective medical records review as the method of investigation, this study was limited by the omissions of information regarding the DNR discussion between patients/surrogate decision-makers and healthcare professionals.

Third, although we have controlled for confounding variables using multivariate logistic regression analysis, there is a possibility that other potential confounding variables are not included and controlled in the multivariate logistic regression models due to lack of information documented in the medical records.

Finally, this study compared the demographic characteristics of the DNR patients with the non-DNR patients, but we did not know whether the DNR consent was provided directly by the patients or by the surrogate decision-makers.

## CONCLUSION

According to our study, old age, an admission diagnosis of “nonoperative, cardiac failure/insufficiency,” and severe acute illness at SICU admission were significantly associated with an increasing likelihood of consenting to a DNR order. We also found that the religious background of Buddhism/Daoism was associated with a lower likelihood of consenting to a DNR order. The result will hopefully raise the awareness that patients’ religious background of Buddhism/Daoism may play a role in the decision-making process of consenting to a DNR order. Furthermore, poor social support may be associated with more likelihood of consenting to a DNR order. However, our study does not intend to presume that the patients with poor social support, or the religious background of Buddhism/Daoism, always have a consistent preference to consent to a DNR order. Instead, carefully deliberating the patient's preference with regard to DNR should always be highlighted regardless of the patient's religious background and social support as indicated by work and marital status.

## References

[1] Standards for cardiopulmonary resuscitation (CPR) and emergency cardiac care (ECC). V. Medicolegal considerations and recommendations. *JAMA* 1974; 227 (Suppl):864–868. 4405914

[2] Optimum care for hopelessly ill patients. A report of the Clinical Care Committee of the Massachusetts General Hospital. *N Engl J Med* 1976; 295:362–364. 934224 10.1056/NEJM197608122950704

[3] Rabkin MT Gillerman G Rice NR Orders not to resuscitate. *N Engl J Med* 1976; 295:364–366. 934225 10.1056/NEJM197608122950705

[4] President's Commission for the Study of Ethical Problems in Medicine and Biomedical and Behavioral Research. Deciding to Forego Life-Sustaining Treatment: Ethical, Medical, and Legal Issues in Treatment Decisions.: Washington, DC: Government Printing Office; 1983

[5] The Joint Commission.1994 Accreditation Manual for Hospitals. Volume I: Standards Illinois: Oakbrook Terrace: 1994

[6] Brizzi M Abul-Kasim K Jalakas M Early do-not-resuscitate orders in intracerebral haemorrhage; frequency and predictive value for death and functional outcome. A retrospective cohort study. *Scand J Trauma Resusc Emerg Med* 2012; 20:36. 22632100 10.1186/1757-7241-20-36 PMC3487937

[7] Chen YY Gordon NH Connors AF Jr Factors associated with two different protocols of do-not-resuscitate orders in a medical ICU^∗^. *Crit Care Med* 2014; 42:2188–2196. 24810524 10.1097/CCM.0000000000000411

[8] Meilink M van de Wetering K Klip H Discussing and documenting (do not attempt) resuscitation orders in a Dutch Hospital: a disappointing reality. *Resuscitation* 2006; 71:322–326. 17064837 10.1016/j.resuscitation.2006.05.013

[9] Tschann JM Kaufman SR Micco GP Family involvement in end-of-life hospital care. *J Am Geriatr Soc* 2003; 51:835–840. 12757572 10.1046/j.1365-2389.2003.51266.x

[10] Sinuff T Cook DJ Rocker GM DNR directives are established early in mechanically ventilated intensive care unit patients. *Can J Anaesth* 2004; 51:1034–1041. 15574557 10.1007/BF03018494

[11] Nathens AB Rivara FP Wang J Variation in the rates of do not resuscitate orders after major trauma and the impact of intensive care unit environment. *J Trauma* 2008; 64:81–88. 18188103 10.1097/TA.0b013e31815dd4d7

[12] Mack JW Paulk ME Viswanath K Racial disparities in the outcomes of communication on medical care received near death. *Arch Intern Med* 2010; 170:1533–1540. 20876403 10.1001/archinternmed.2010.322 PMC3739279

[13] Saager L Kurz A Deogaonkar A Pre-existing do-not-resuscitate orders are not associated with increased postoperative morbidity at 30 days in surgical patients. *Crit Care Med* 2011; 39:1036–1041. 21336133 10.1097/CCM.0b013e31820eb4fc

[14] Henneman EA Baird B Bellamy PE Effect of do-not-resuscitate orders on the nursing care of critically ill patients. *Am J Crit Care* 1994; 3:467–472. 7834009

[15] Beach MC Morrison RS The effect of do-not-resuscitate orders on physician decision-making. *J Am Geriatr Soc* 2002; 50:2057–2061. 12473020 10.1046/j.1532-5415.2002.50620.x

[16] Hinds PS Schum L Baker JN Key factors affecting dying children and their families. *J Palliat Med* 2005; 8 Suppl 1:S70–78. 16499471 10.1089/jpm.2005.8.s-70

[17] Cohen RI Lisker GN Eichorn A The impact of do-not-resuscitate order on triage decisions to a medical intensive care unit. *J Crit Care* 2009; 24:311–315. 19327284 10.1016/j.jcrc.2008.01.007

[18] Park YR Kim JA Kim K Changes in how ICU nurses perceive the DNR decision and their nursing activity after implementing it. *Nurs Ethics* 2011; 18:802–813. 21974942 10.1177/0969733011410093

[19] Karches KE Chung GS Arora V Religiosity, spirituality, and end-of-life planning: a single-site survey of medical inpatients. *J Pain Symptom Manage* 2012; 44:843–851. 22727947 10.1016/j.jpainsymman.2011.12.277 PMC3459155

[20] Jaul E Zabari Y Brodsky J Spiritual background and its association with the medical decision of, DNR at terminal life stages. *Arch Gerontol Geriatr* 2014; 58:25–29. 24029615 10.1016/j.archger.2013.08.004

[21] Hileli I Weyl Ben Arush M Hakim F Association between religious and socio-economic background of parents of children with solid tumors and DNR orders. *Pediatr Blood Cancer* 2014; 61:265–268. 23940107 10.1002/pbc.24712

[22] Chiu TY Hu WY Huang HL Prevailing ethical dilemmas in terminal care for patients with cancer in Taiwan. *J Clin Oncol* 2009; 27:3964–3968. 19470919 10.1200/JCO.2008.21.4643

[23] Liu JM Lin WC Chen YM The status of the do-not-resuscitate order in Chinese clinical trial patients in a cancer centre. *J Med Ethics* 1999; 25:309–314. 10461593 10.1136/jme.25.4.309 PMC479239

[24] Chang Y Huang CF Lin CC Do-not-resuscitate orders for critically ill patients in intensive care. *Nurs Ethics* 2010; 17:445–455. 20610578 10.1177/0969733010364893

[25] Huang YC Huang SJ Ko WJ Survey of do-not-resuscitate orders in surgical intensive care units. *J Formos Med Assoc* 2010; 109:201–208. 20434028 10.1016/S0929-6646(10)60043-5

[26] Sulmasy DP Spirituality, religion, and clinical care. *Chest* 2009; 135:1634–1642. 19497898 10.1378/chest.08-2241

[27] Cullen DJ Civetta JM Briggs BA Therapeutic intervention scoring system: a method for quantitative comparison of patient care. *Crit Care Med* 1974; 2:57–60. 4832281

[28] Knaus WA Draper EA Wagner DP APACHE II: a severity of disease classification system. *Crit Care Med* 1985; 13:818–829. 3928249

[29] Hanley JA McNeil BJ The meaning and use of the area under a receiver operating characteristic (ROC) curve. *Radiology* 1982; 143:29–36. 7063747 10.1148/radiology.143.1.7063747

[30] Lemeshow S Hosmer DW Jr A review of goodness of fit statistics for use in the development of logistic regression models. *Am J Epidemiol* 1982; 115:92–106. 7055134 10.1093/oxfordjournals.aje.a113284

[31] Phillips RS Wenger NS Teno J Choices of seriously ill patients about cardiopulmonary resuscitation: correlates and outcomes. *Am J Med* 1996; 100:128–137. 8629646 10.1016/s0002-9343(97)89450-8

[32] The Global Religious Landscape: A Report on the Size and Distribution of the World's Major Religious Groups as of 2010. Pew Research Center; 2012

[33] http://en.wikipedia.org/wiki/Religion_in_Taiwan.

[34] Tang ST Liu TW Lai MS Concordance of preferences for end-of-life care between terminally ill cancer patients and their family caregivers in Taiwan. *J Pain Symptom Manage* 2005; 30:510–518. 16376737 10.1016/j.jpainsymman.2005.05.019

[35] Bailey FA Allen RS Williams BR Do-not-resuscitate orders in the last days of life. *J Palliat Med* 2012; 15:751–759. 22536938 10.1089/jpm.2011.0321

[36] Cardenas-Turanzas M Gaeta S Ashoori A Demographic and clinical determinants of having do not resuscitate orders in the intensive care unit of a comprehensive cancer center. *J Palliat Med* 2011; 14:45–50. 21194303 10.1089/jpm.2010.0165

[37] Chen YY Chen L Huang TS Significant social events and increasing use of life-sustaining treatment: trend analysis using extracorporeal membrane oxygenation as an example. *BMC Med Ethics* 2014; 15:21. 24592981 10.1186/1472-6939-15-21 PMC3975881

[38] http://en.wikipedia.org/wiki/Filial_piety.

[39] Cong Y Ethical challenges in critical care medicine: a Chinese perspective. *J Med Philos* 1998; 23:581–600. 10190842 10.1076/jmep.23.6.581.2558

[40] Chen RC The Spirit of Humanism in Terminal Care. Taiwan Experience. *Open Area Stud J* 2009; 2:7–11.

[41] Kwok T Twinn S Yan E The attitudes of Chinese family caregivers of older people with dementia towards life sustaining treatments. *J Adv Nurs* 2007; 58:256–262. 17474914 10.1111/j.1365-2648.2007.04230.x

[42] Bulow HH Sprung CL Reinhart K The world's major religions’ points of view on end-of-life decisions in the intensive care unit. *Intensive Care Med* 2008; 34:423–430. 18157484 10.1007/s00134-007-0973-8

[43] McCormick AJ Buddhist ethics and end-of-life care decisions. *J Soc Work End Life Palliat Care* 2013; 9:209–225. 23777235 10.1080/15524256.2013.794060

[44] http://en.wikipedia.org/wiki/Karma.

[45] Bowman KW Hui EC Bioethics for clinicians: 20. Chinese bioethics. *CMAJ* 2000; 163:1481–1485. 11192658 PMC80420

[46] Uchino BN Social support and health: a review of physiological processes potentially underlying links to disease outcomes. *J Behav Med* 2006; 29:377–387. 16758315 10.1007/s10865-006-9056-5

[47] Mookadam F Arthur HM Social support and its relationship to morbidity and mortality after acute myocardial infarction: systematic overview. *Arch Intern Med* 2004; 164:1514–1518. 15277281 10.1001/archinte.164.14.1514

[48] Fratiglioni L Paillard-Borg S Winblad B An active and socially integrated lifestyle in late life might protect against dementia. *Lancet Neurol* 2004; 3:343–353. 15157849 10.1016/S1474-4422(04)00767-7

[49] Huang CH Hu WY Chiu TY The practicalities of terminally ill patients signing their own DNR orders: a study in Taiwan. *J Med Ethics* 2008; 34:336–340. 18448711 10.1136/jme.2007.020735

[50] Wen KY Lin YC Cheng JF Insights into Chinese perspectives on do-not-resuscitate (DNR) orders from an examination of DNR order form completeness for cancer patients. *Support Care Cancer* 2013; 21:2593–2598. 23653012 10.1007/s00520-013-1827-2 PMC3728434

